# Noninvasive 11.7‐T Magnetic Resonance Spectroscopy and Imaging Reveals Retinal Metabolic Alterations Induced by Blue Light Exposure

**DOI:** 10.1002/nbm.70240

**Published:** 2026-02-16

**Authors:** Lacramioara Samoila, Alexandru Farcasanu, Simion Simon, Ede Bodoki, Ovidiu Samoila, Oliviu Vostinaru, Elena Dinte, Andreea Elena Bodoki, Dan Iudean, Calin Muresan, Simona Clichici

**Affiliations:** ^1^ Department of Physiology “Iuliu Hațieganu” University of Medicine and Pharmacy Cluj‐Napoca Romania; ^2^ Institute for Interdisciplinary Research in Bio‐Nano‐Science, Preclinical MRI Center (INSPIRE Platform) Babeș‐Bolyai University Cluj‐Napoca Romania; ^3^ Amethyst Radiotherapy Center Cluj‐Napoca Romania; ^4^ Department of Analytical Chemistry “Iuliu Hațieganu” University of Medicine and Pharmacy Cluj‐Napoca Romania; ^5^ Department of Ophthalmology “Iuliu Hațieganu” University of Medicine and Pharmacy Cluj‐Napoca Romania; ^6^ Department of Pharmacology, Physiology and Physiopathology “Iuliu Hațieganu” University of Medicine and Pharmacy Cluj‐Napoca Romania; ^7^ Department of Pharmaceutical Technology and Biopharmaceutics “Iuliu Hațieganu” University of Medicine and Pharmacy Cluj‐Napoca Romania; ^8^ General and Inorganic Chemistry Department “Iuliu Hațieganu” University of Medicine and Pharmacy Cluj‐Napoca Romania; ^9^ Faculty of Electrical Engineering, Department of Electrotechnics and Measurements Technical University Cluj‐Napoca Romania

**Keywords:** age‐related macular degeneration, blue light, magnetic resonance spectroscopy, retina

## Abstract

The eye is a complex structure, with multiple systems involved in focusing and detecting light. Among them, the retina, an integral component of the central nervous system, is considered the most vital and exhibits the highest metabolic activity among all tissues in the human body. It interacts with light, and excessive exposure, especially to blue light, is prone to produce degeneration, mainly through oxidative reactions. This mechanism is involved in age‐related macular degeneration or diabetic retinopathy. Animal research is important, considering the high prevalence of these diseases; yet noninvasive procedures involving this research are lacking so far. Our objective was to apply an animal model of oxidative stress and monitor the metabolic changes using 11.7‐T ^1^H‐magnetic resonance spectroscopy (^1^H‐MRS). We exposed adult rats to high intensity blue light, at 440 nm (6000 lx) and investigated retinal metabolic changes up to 48 h post exposure. The acquired spectrum highlighted the presence of several essential retinal metabolites, including lipids (alkyl chain CH_2_), lactate, *N*‐acetylaspartate (NAA), glutamate (Glu), choline (Cho), taurine (Tau), creatine (Cre), and glucose (Glc). Blue light induced specific changes, relatable to oxidative stress, and ^1^H‐MRS allowed us to follow the dynamic metabolic changes post exposure. This is the first in vivo spectroscopic study of the retinal tissue in which no animals were sacrificed. To validate the in vivo metabolite assignments, localized ex vivo ^1^H‐MRS was performed on eyes from separate animals that had not been exposed to blue light.

## Graphical abstract

Retinal metabolic changes were analyzed using proton magnetic resonance spectroscopy (^1^H‐MRS), marking an initial application of this non‐invasive method on living eyes. Key metabolites were identified within the retinal region of adult rats. The observed changes following intense blue light exposure were predominantly attributable to the retina, as it is the sole tissue exhibiting high reactivity to light.

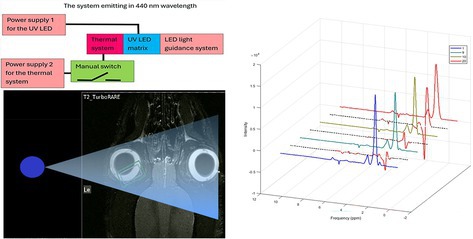

**Abbreviations**
^1^H‐MRS: ^1^H‐magnetic resonance spectroscopy
AMD: age‐related macular degeneration
Cho: choline
Cre: creatine
Gln: glutama
Glc: glucose
Glu: glutamate
jMRUI: Java Magnetic Resonance User Interface
Lac: lactate
mI: myo‐inositol
MRI: magnetic resonance imaging
MRS: magnetic resonance spectroscopy
NAA: *N*‐acetylaspartate
ROI: region of interest
RPE: retinal pigment epithelium
Tau: taurine
tCho: total choline
TE: time of echo

## 1 Introduction

The retina is one of the most sensitive structures to light, and excessive exposure to blue light (400–500 nm) is suspected of producing oxidative stress and metabolic alterations. Aging retina is particularly susceptible to such alterations, with the consequence of accelerated retinal degeneration. Almost all people over the age of 65 will have extracellular lipid and protein accumulation beneath the retina, with inflammation and complement activation that will progress in many cases towards a vision‐threatening disease, age‐related macular degeneration (AMD). The association between oxidative stress and inflammation is not limited to AMD; many other eye conditions have this link in their pathogenetic chain: diabetic retinopathy, retinal dystrophies, or even cataracts.

AMD is the main cause for loss of vision in the elderly. It is a multifactorial disease, with the involvement of both genetic and environmental factors. AMD can be investigated with specific ophthalmic tools (e.g., ocular coherence tomography) only after the disease produces significant retinal alterations. Noninvasive functional/metabolic methods in vivo for the early detection of these retinal changes are insufficient. Electrophysiology studies allow an indirect assessment of functional changes in retinas, with the reduction of *a* and *b* waves after light exposures. Retinal changes in animal models can usually be assessed only through invasive methods, especially histology and immunohistochemistry, meaning the sacrifice of the animal. There is a link between metabolic and functional changes that would be interesting to explore, from the earliest phases possible, allowing for better understanding of AMD.

Animal retinal degeneration models that simulate human retinal diseases are well known, especially in rodents [1]. Rodent retinas lack a proper macula, but the processes involving retinal function are very similar to humans. Degeneration models include models of oxidative stress (superoxide dismutase knockout variants and cigarette smoke/hydroquinone exposure), models of inflammation (complement factor H knockout and transgenic mice overexpressing C3), multigenetic variants, streptozotocin (inducing hyperglycemia), laser‐induced neovascularization, or phototoxicity models [2]. Different light sources are used in the phototoxicity models, resulting in different wavelengths of exposure (usually white light or blue light) and various intensities of light (as low as 150 lx). In vivo studies have well documented the consequences of retinal exposure to blue light, with light‐emitting diode (LED) sources. At high intensities, it is expected to have metabolic changes in the early phase, followed later by retinal thinning, especially affecting the photoreceptor layer and retinal pigment epithelium (RPE).

**FIGURE 1:**
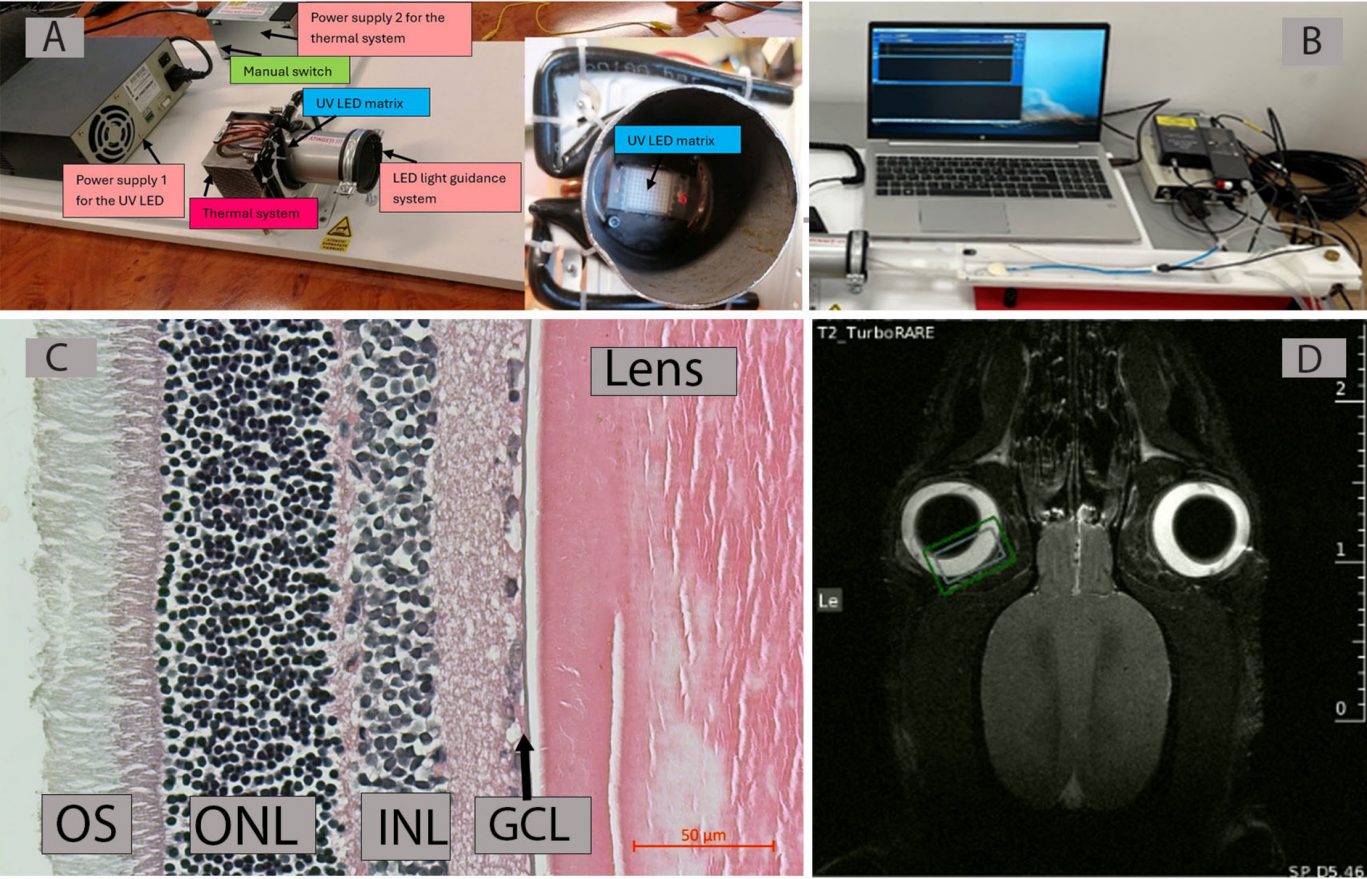
(A) Schematic of the blue light–emitting device, with the UV‐LED matrix, emitting at 440 nm. (B) Tray for animal (rat) placement and monitoring in front of the blue light–emitting assembly, custom design, and computer controlled. (C) Histological view of rat retina (hematoxylin–eosin). GCL—ganglion cell layer, INL—inner nuclear layer, Lens—crystalline lens, ONL—outer nuclear layer, OS—photoreceptors' outer segments (S.O. personal collection, unpublished). (D) MRI‐scanned volumes in the right eye (gray rectangle—^1^H‐MRS voxel placement, green rectangle—magnetic field homogenization area).

Magnetic resonance imaging (MRI) is a noninvasive modality that allows a good representation of the anatomy and function of in vivo structures. When assessing the retina, the limitations of conventional MRI do not allow a complete investigation of this neural layer, measuring approximately 200 μm, nor the metabolic changes that occur physiologically or after oxidative stress. In rats, half of the retina thickness is occupied by the photoreceptors, with body (outer nuclear layer) and external segments (Figure 1C). A combination of MRI with spectroscopy would allow the analysis of chemical composition of the retina, given that the low spatial resolution of MRI could be improved. Magnetic resonance spectroscopy (MRS) has been used in analyzing other organs, both in humans and animals: usually brain, but also heart, skeletal muscle [3] or liver [4]. Proton MRS (^1^H‐magnetic resonance spectroscopy [^1^H‐MRS]) was the main acquisition.

Recent advances in in vivo MRS have significantly improved the field—through standardized reporting, advanced spectral editing, and high‐resolution MRS imaging—enhancing the ability to detect and quantify brain metabolites relevant for both research and clinical applications [5].

Through combinations of edited sequences (e.g., MEGA‐PRESS/HERMES), higher magnetic fields, and modern quantification pipelines, reliable estimates of *N*‐acetylaspartate (NAA), total creatine (Cre), myo‐inositol (mI), glutamate (Glu), Glutamine (Gln), gamma‐aminobutyric acid (GABA), and compounds such as glutathione can be obtained; however, Glu/Gln separation and corrections for macromolecular signals remain methodological challenges [6].

Recently, high‐resolution MRS techniques with short acquisition times, such as fast high‐resolution metabolite mapping in the rat brain using proton‐free induction decay–magnetic resonance spectroscopic imaging (^1^H‐FID‐MRSI) at 14.1 T, have demonstrated the feasibility of local metabolite mapping even in the rat brain using ultrahigh fields—highlighting the potential of advanced methods for metabolic studies in small tissues [7].

In conclusion, next‐generation in vivo MRS provides robust tools for noninvasive characterization of cerebral and tumor metabolism. Nevertheless, quantitative and biological interpretation of composite signals (particularly total choline [tCho]) requires standardized protocols, multimodal validation, and careful consideration of technical limitations [8].

### 1.1 Purpose of the Study

Acute exposure of the eye to blue light in adult rats induces metabolic changes, which so far have been investigated using invasive methods that require animal sacrifice. Our hypothesis is that these changes can be detected using ^1^H‐MRS, a noninvasive technique that does not require the sacrifice of the animals involved in the experiments. Because there are no published references regarding localized MRS of the rat retina, a localized ex vivo ^1^H‐MRS experiment was also performed on eyes from separate animals, which were sacrificed for this purpose and had not been exposed to blue light, in order to validate the metabolite assignments obtained in vivo. To the best of our knowledge, no published data exist regarding the application of MRS to ocular structures, in vivo.

## 2 Materials and Methods

### 2.1 Animal Model

#### 2.1.1 Animal Care and Use

Male Wistar rats with an average body weight of approximately 220 g were used in this study. The animals were provided by the Center for Experimental Medicine of the “Iuliu Hațieganu” University of Medicine and Pharmacy in Cluj‐Napoca, Romania. The animals were maintained under standard laboratory conditions: ambient temperature of 23°C ± 1°C, relative humidity of 55% ± 5%, and a 12‐h light/dark cycle. They were fed standard rodent pellet food, and water was available ad libitum.

All experimental procedures were conducted in accordance with the provisions of Directive 2010/63/EU on the protection of animals used for scientific purposes and the applicable national legislation (Law No. 43/2014). The study protocol was approved by the National Sanitary Veterinary and Food Safety Authority (ANSVSA), under Project Authorization No. 414/20.08.2024.

Animal handling procedures, especially those involving potential pain or discomfort, were performed following the recommendations of Directive 2010/63/EU, with the goal of minimizing suffering and ensuring animal welfare throughout the experimental period.

#### 2.1.2 Exposure Procedure and MRI Data Acquisition

During blue light exposure and MRI data acquisition, the rats were anesthetized with isoflurane at a concentration of 1.5%–2.5%. The body temperature of the animals was maintained at 37.5°C ± 1.0°C using circulating warm water, and it was monitored via a rectal thermosensor. Respiratory rate and body temperature were continuously monitored using a small animal monitoring system (SA Instruments, New York, NY, USA).

### 2.2 Blue Light Exposure Device

The system used to expose the animals to blue light was composed of two distinct power supplies, each serving a specific operational function (Figure 1A). The primary power source was a variable supply designed to regulate the intensity of a UV‐LED matrix having a wavelength of 440 nm (±3%), working at 100 W, between 30 and 34 V at 3000 mA, thereby enabling fine control over the emitted light output. The secondary power supply was allocated to the thermal management subsystem, which maintained the LEDs within optimal temperature thresholds to ensure performance stability and component longevity. An integrated manual switch allowed for independent activation or deactivation of the cooling mechanism. The blue light radiation was guided and concentrated through an aluminum tube, which facilitated directional exposure onto a targeted surface, enhancing the system's precision and efficacy in applications requiring controlled illumination.

The system allowed controlled illumination with blue light for a duration of 15 min. The intensity of light was verified before each exposure at the end of the tube, where the animal's head was placed, with a luxmeter (BK Precision), confirming a 6000‐lx dosage. The animal was placed on a tray that allowed monitoring and horizontal/vertical positioning with the eye facing the source (Figure 1B).

### 2.3 Acquisition of ^1^H‐MRS

The MRI experiment was performed using a Bruker BioSpec 11.7‐T system (Ettlingen, Germany), equipped with a horizontal bore scanner for small animals and a BGA‐9S gradient system (maximum gradient strength of 740 mT/m). For radiofrequency (RF) transmission, an FRES5001H089/072QUadTOAD probe with an active diameter of 60 mm was used. For reception, the surface RF coil SUC 500 1H R.BR. QSN RO AD was employed, using the Bruker ParaVision 360 V3.5 interface.

During the investigation, the animals were anesthetized with isoflurane (1%) and oxygen in a 1:3 ratio, delivered via a Dräger Vapor 2000 vaporizer. Their physiological status (ECG, respiration, and body temperature) was monitored using a surveillance system, and their temperature was maintained using a heated pad.

Two‐dimensional (2D) coronal images were acquired using a spin echo rapid acquisition with relaxation enhancement (RARE) protocol, with a repetition time (TR) = 2500 ms and an echo time (TE) = 31.5 ms. The acquisition matrix was 256 × 256 pixels, within a field of view (FOV) of 39.2 × 35 mm, resulting in an image resolution of 0.153 × 0.137 mm.

A second 2D imaging protocol was also employed, consisting of axial images acquired using a gradient echo fast low‐angle shot (FLASH) sequence, with a TR = 200 ms, TE = 3 ms, and a flip angle (FA) = 25°. The acquisition matrix was 512 × 512 pixels within a 35 × 35 mm FOV, resulting in an image resolution of 0.068 × 0.068 mm. These two sets of 2D images were used for voxel positioning for localized spectroscopy.

The ^1^H‐MRS protocol used was point resolved spectroscopy (PRESS), with a TR = 1800 ms and a TE = 16.5 ms. The voxel of interest had dimensions of 0.98 × 4.68 × 4.87 mm (Figure 1D). To suppress the water signal from the region of interest (ROI), the VAPOR 1 protocol was applied. For the ex vivo investigations, the same acquisition settings were maintained to ensure consistency, allowing direct comparison with the in vivo measurements.

### 2.4 MATLAB Processing and Analysis

#### 2.4.1 Custom Script

- Reading and aligning spectra.
- Calculation of differences: intensityDiff = spectraData{i + 1}(:,2) − spectraData{i}(:,2).
- 3D plotting for visualization of evolution over time.
- Black dotted line = total difference.
- Red line = significant percentage changes with a threshold of 2%.
- Allows manual selection of metabolic peaks.
- Normalizes all spectra to the water peak.
- Extracts the temporal evolution of the selected peaks.
- Computes kinetic rates.
- Generates a final table with all kinetic parameters.
- Plots the overlaid spectra in 2D.

#### 2.4.2 Temporal Differential Spectral Analysis

To evaluate metabolic changes over time in the retina, we used a computational method of differential analysis of MRI spectra, implemented in a MATLAB [9] script. This approach involves the conversion of frequencies into chemical units (ppm) and the three‐dimensional representation of successive spectra as a function of time, allowing visual observation of the evolution of metabolic signals.

The differences between the successive spectra were calculated punctually, according to the relationship:

$$\Delta I_{i}\left(f\right)=I_{i+1}\left(f\right)-I_{i}\left(f\right)$$

where *I*_*i*_(*f*) represents the signal strength at frequency *f* at time *t*_*i*_. This simple but robust approach allows for the isolation of significant signal changes that may indicate the emergence or disappearance of specific metabolites. A positive signal (Δ*I* > 0) reflects a local increase in concentration, while a negative signal (Δ*I* < 0) indicates a decrease. This method provides increased sensitivity to subtle but systematic variations in signal strength over time, allowing the detection of metabolic changes without requiring predefined spectral patterns.

Differences are plotted with black dotted lines (for total difference) and solid red lines for significant changes. In addition, the 3D representation of spectra provides an intuitive advantage in the visual analysis of dynamic processes.

In addition, metabolite peaks of interest were manually selected on representative spectra, and the water signal was used as a reference for normalization. Normalized intensities were then tracked over time to generate kinetic curves for each metabolite. From these curves, both instantaneous rates between consecutive time points and the overall average rate were calculated, providing a quantitative assessment of temporal metabolic changes.

By combining differential spectral analysis with water‐normalized kinetic curves, this approach allows a sensitive and rigorous evaluation of dynamic biochemical processes, facilitating the correlation of spectral variations with relevant physiological parameters in in vivo studies.

By using this method, we aimed at detailed temporal characterization of local biochemical processes, with the potential to correlate spectral changes with relevant physiological parameters during in vivo studies.

### 2.5 Simulation of Metabolites With Java Magnetic Resonance User Interface (jMRUI)

To identify and quantify the metabolic components contributing to the MR signal, spectral data were processed using the jMRUI software [10]. In particular, the AMARES module [11], a nonlinear least‐squares (NLLS) quantification algorithm, was employed to deconvolute the broad overlapping signals observed in the in vivo rat eye. AMARES allows precise separation and quantification of superimposed peaks, providing reliable metabolite parameters, including amplitude, frequency, linewidth, and phase. This approach enables rigorous signal assignment and accurate interpretation of in vivo spectra, overcoming the limitations of conventional integration methods and ensuring high fidelity in metabolite analysis.

### 2.6 Ex Vivo MRS Study

For the ex vivo experiments, separate animals were sacrificed, and the eyes were harvested with the preservation of at least 1‐cm optic nerve for good orientation. The eyes were then mounted in Eppendorf tubes under distilled water. Acquisition of ^1^H‐MRS was performed with the same method described above.

## 3 Results

### 3.1 Pre‐Exposure ^1^H Spectra Across Different Subjects: Assessment of Interindividual Reproducibility

**FIGURE 2:**
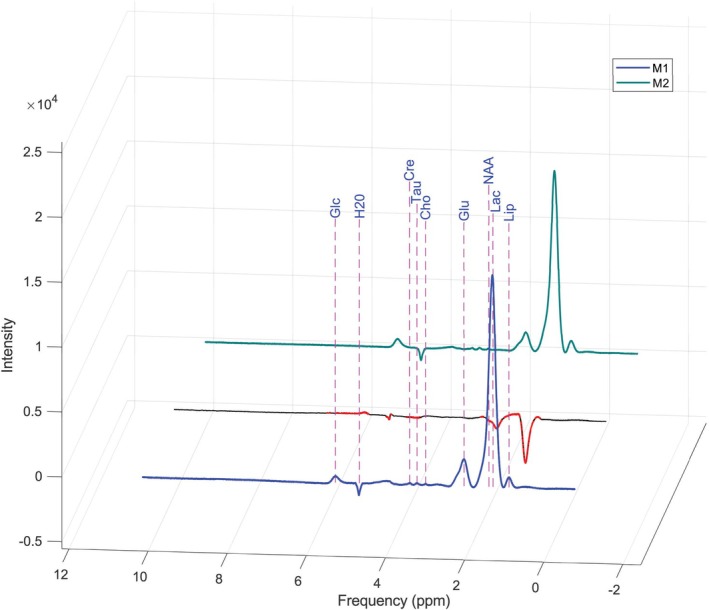
Pre‐exposure ^1^H spectra across different subjects: assessment of interindividual reproducibility. Blue and green spectra correspond to different subjects, while the red/black line highlights the differences between the two (black line where there are no differences and red line where the difference exceeds 2%). Cho—choline, Cre—creatine, Glc—glucose, Glu—glutamate, H_2_O—water, Lac—lactate, Lip—lipids, NAA—*N*‐acetylaspartate, Tau—taurine.

The initial analyses of the ^1^H‐MRS spectra were performed with the aim of evaluating the reproducibility of the signals obtained across individual subjects. To this end, spectra recorded from rats prior to experimental exposure were compared. Figure 2 presents two representative spectra: The spectrum shown in blue corresponds to one subject, while the green spectrum corresponds to a second subject. The dotted line represents the difference between the two spectra, and the segment highlighted with a continuous red line indicates the region where the difference exceeds 2%. The resulting signal is stable among the individuals, with only slight variations in the peaks of lactate and Glu metabolites.

To allow a coherent comparison of signal intensities, the intensity of the tissue water signal was used as an internal reference. This approach enabled standardized normalization of data across subjects. The differences observed between the spectra of the two individuals were minimal and supported the reproducibility and consistency of the method used for spectral acquisition.

The ^1^H‐MRS spectra obtained from rat retinal tissue exhibit broad peaks due to the semisolid nature of the tissue and its high‐water content, which influences the local magnetic environment. In semisolid tissues such as the retina, molecular motion is restricted, leading to static dipolar interactions and chemical shift anisotropy that cannot be rapidly averaged by molecular motion. These interactions cause increased resonance frequency dispersion and, consequently, significant line broadening in the MRS spectra. Furthermore, the biological heterogeneity of the tissue and the presence of the aqueous environment contribute to local magnetic field variations, further amplifying the line broadening effect. Thus, these physicochemical characteristics of the retinal environment give rise to broad spectral lines characteristic of this class of biological samples [12].

### 3.2 Spectra ^1^H Before and After Exposure

**FIGURE 3:**
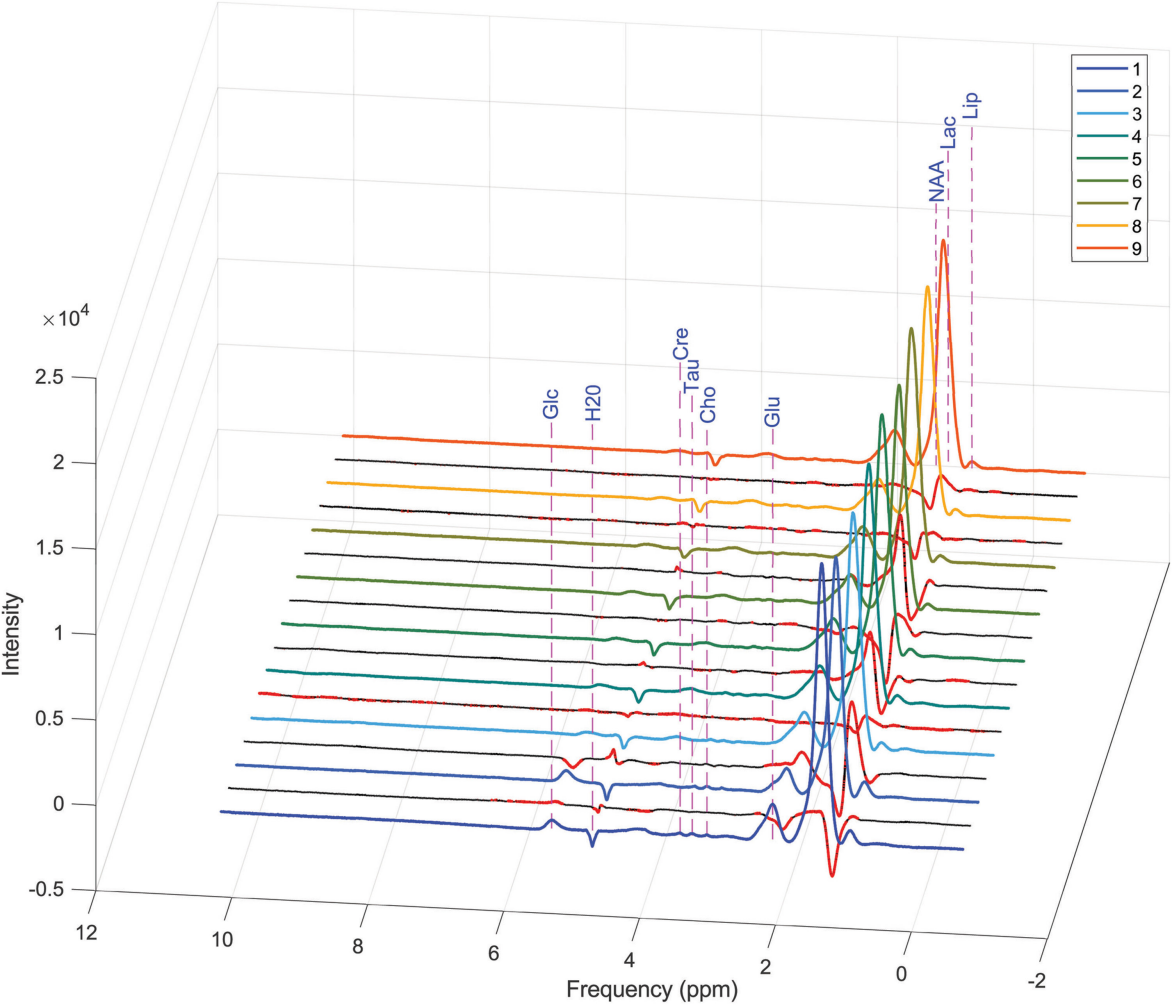
Evolution of the ^1^H‐MRS spectra acquired from the investigated region before and after exposure to blue light. The first spectrum (T1 = 0 min) corresponds to the reference measurement taken prior to exposure, while the subsequent nine spectra were acquired at 10‐min intervals up to 80 min post exposure. The differences between successive investigations are highlighted with the red/black lines (black line where there are no differences and red line where the difference exceeds 2%). Cho—choline, Cre—creatine, Glc—glucose, Glu—glutamate, H_2_O—water, Lac—lactate, Lip—lipids, NAA—*N*‐acetylaspartate, Tau—taurine.

**FIGURE 4:**
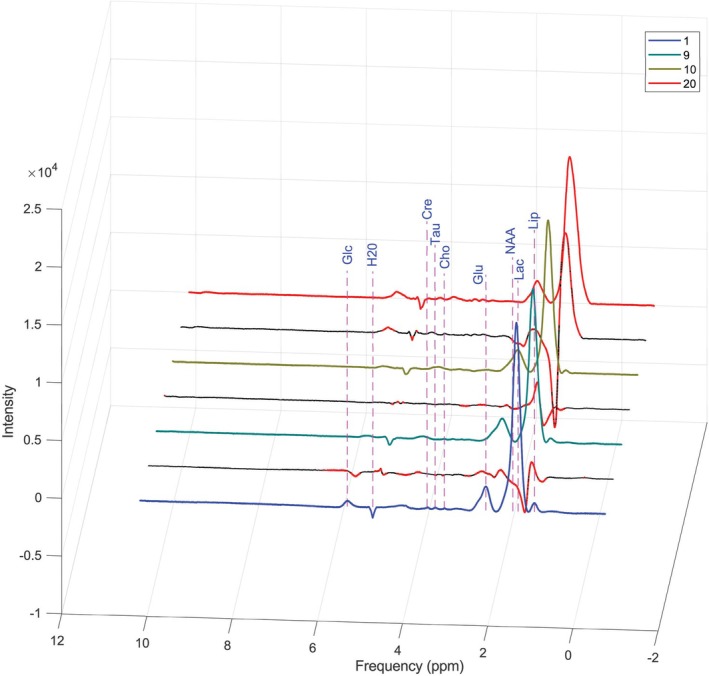
^1^H‐MRS spectra recorded at 24 (Spectrum 10) and 48 h (Spectrum 20) following blue light exposure, compared with the baseline spectrum (Spectrum 1) and 80 min post exposure (Spectrum 9). These measurements were performed to assess potential delayed metabolic alterations. Minor variations in the intensity of certain peaks can be observed (red/black lines between neighboring spectra), suggesting progressive metabolic adaptations over time (black line where there are no differences and red line where the difference exceeds 2%). Cho—choline, Cre—creatine, Glc—glucose, Glu—glutamate, H_2_O—water, Lac—lactate, Lip—lipids, NAA—*N*‐acetylaspartate, Tau—taurine.

The same MATLAB script was applied to the spectra acquired 10 min after exposing the animal to blue light. Spectral acquisitions were subsequently repeated at regular intervals of 10 min over a period of 80 min to assess potential immediate metabolic changes. Figure 3 displays the nine spectra: The first corresponds to the baseline (prior to exposure), while the remaining spectra were acquired post exposure. No significant acute metabolic alterations were observed. To investigate delayed metabolic effects, the experiments were repeated at 24 and 48 h post exposure; the corresponding results are presented in Figure 4. Delayed metabolic effects were observed 48 h post exposure, indicating that certain biochemical alterations require a longer time interval to become detectable by MR spectroscopy.

**FIGURE 5:**
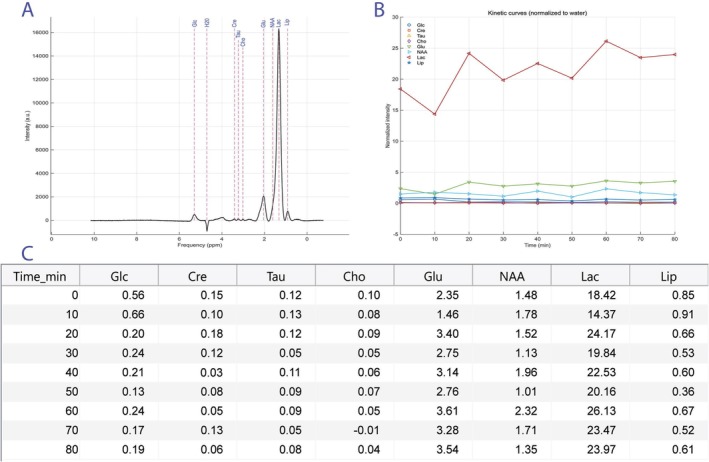
Kinetic curves—comparative analysis of the ^1^H spectra. The kinetic curves shown in Panel B were obtained by quantifying the spectral peaks of metabolites in each spectrum of the ^1^H‐MRS time series. The ppm position of each metabolite was manually defined on the first spectrum (Panel A), and this position was subsequently used as a reference to automatically extract the corresponding intensity from each following spectrum by identifying the closest spectral data point. For each metabolite and each acquisition time, the intensity was computed as the signal amplitude (presented in Panel C—the table below) at the corresponding spectral position. To remove global variations due to sensitivity changes, subject positioning, or instrumental instability, all intensities were normalized to the intensity of the water peak in the same spectrum, according to $I_{\mathrm{norm}}\left(t\right)=\frac{I_{\text{metabolite}}\left(t\right)}{|I_{\text{water}}\left(t\right)|}$, where $I_{\text{metabolite}}\left(t\right)$ is the metabolite intensity at time t and $I_{\text{water}}\left(t\right)$ is the intensity of the water peak at the same time point. The time series was constructed assuming a constant interval of 10 min between acquisitions, so that the displayed time points are 0, 10, 20, …, 80 min. These times correspond exactly to the “Time_min” column in the table shown below. The values plotted in Panel B therefore represent normalized, dimensionless intensities proportional to the relative metabolite concentrations, allowing comparison of metabolic dynamics over time without the influence of global signal fluctuations. Cho—choline, Cre—creatine, Glc—glucose, Glu—glutamate, H_2_O—water, Lac—lactate, Lip—lipids, NAA—*N*‐acetylaspartate, Tau—taurine.

The eight processed ^1^H‐NMR spectra revealed clear metabolic alterations (Figure 5). The lipid signals (1.03 ppm) showed an increase, and lactate (1.14 ppm) exhibited a marked elevation, indicating enhanced anaerobic metabolism. NAA (1.87 ppm) displayed an abrupt and sustained decrease, suggesting compromised neuronal integrity. Glu (2.3 ppm) showed an initial decline followed by an increase, reflecting a biphasic metabolic response.

For choline (Cho) (3.21 ppm), taurine (3.40 ppm), and Cre (3.81 ppm), no significant variations were observed. Glucose (5.35 ppm) showed a decrease followed by a partial recovery, indicating an initially heightened consumption. The 3D spectra and difference analyses confirmed these trends, and the kinetic rate calculations allowed quantification of the changes, which were compiled in the final table automatically generated by the script.

### 3.3 Ex Vivo Investigations for the Validation of Metabolite Assignments

**FIGURE 6:**
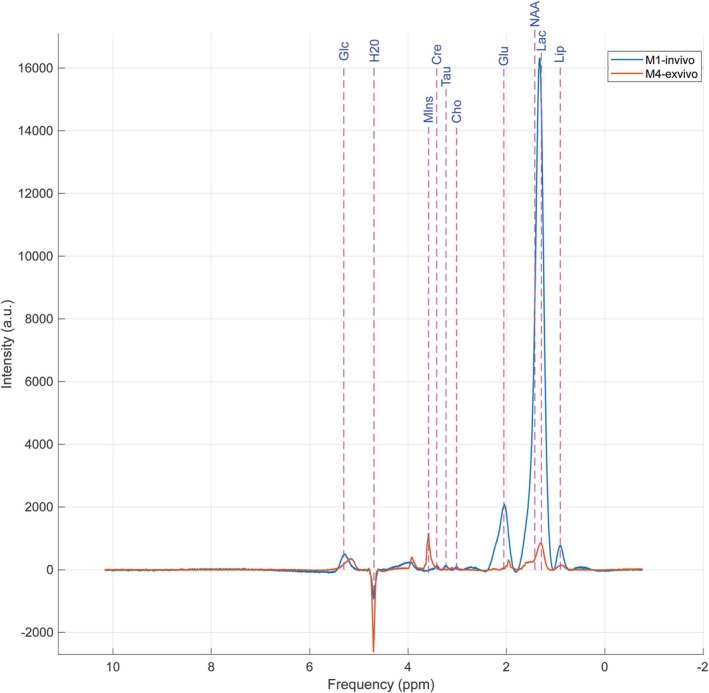
Comparison between acquired spectra in vivo (M1—green) and ex vivo (M2—orange). Cho—choline, Cre—creatine, Glc—glucose, Glu—glutamate, H_2_O—water, Lac—lactate, Lip—lipids, NAA—*N*‐acetylaspartate, Tau—taurine.

Due to the lack of bibliographic references specifically describing the ^1^H‐MRS profiles of retinal tissue, an ex vivo experiment was required to confirm the metabolite assignments identified in vivo. For this purpose, localized ex vivo ^1^H‐MRS measurements were performed on isolated rat eyes that had not been exposed to blue light. The eyes were immersed in distilled water to ensure stable positioning during acquisition and to minimize susceptibility artifacts at the tissue–air interface. In Figure 6, the in vivo spectrum obtained from the animal prior to exposure and the ex vivo spectrum from the extracted eye immersed in distilled water are presented.

The ^1^H‐MRS spectra obtained ex vivo from rat retinal tissue exhibited superior resolution compared with the in vivo spectra, due to the elimination of motion artifacts and the reduction of background noise. This higher resolution allowed the identification and assignment of metabolites, including lactate, NAA, Cre, Cho, mI, and taurine [13]. The mI signal was more prominent ex vivo, likely as a result of the release of this compound from cellular compartments and the reduction of relaxation effects and overlap with other signals, thus supporting and validating the assignment of metabolites detected in vivo [14]. Chemical shifts and peak shapes remained consistent, with the main differences being quantitative rather than qualitative.

### 3.4 Validation of jMRUI

**FIGURE 7:**
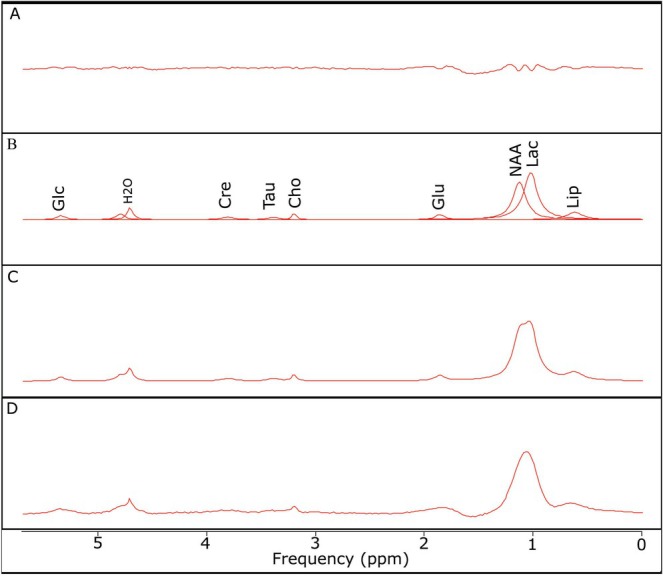
Representative ^1^H‐MRS spectrum illustrating the result of spectral deconvolution using the AMARES algorithm. Residue—the difference between the original signal and the sum of the individual components, highlighting noise and any model mismatches (A); individual components—fitted signals of the individual metabolites overlaid on the acquired signal, showing the contribution of each component to the total spectrum (B); estimate—the sum of all fitted components, representing the reconstructed model of the spectrum (C); and original—the experimentally acquired signal (D). Cho—choline, Cre—creatine, Glc—glucose, Glu—glutamate, H_2_O—water, Lac—lactate, Lip—lipids, NAA—*N*‐acetylaspartate, Tau—taurine.

Subsequently, these spectra were processed in jMRUI software using the AMARES algorithm to simulate and deconvolve the total signal into its metabolic components. This simulation enabled the identification of 10 major metabolites present in the investigated region. Figure 7 illustrates the result of simulating a typical spectrum, and Table 1 presents the chemical shifts of the identified components along with the assignment of each spectral signature to its corresponding metabolite. The acquired MR spectrum highlights the presence of several essential metabolites, including lipids (particularly alkyl chain CH_2_—methylene), lactate, NAA, Glu, Cho, taurine, Cre, water (H_2_O), and glucose.

**TABLE 1:** List of chemical shifts (in ppm) and corresponding assignments for each identified metabolite. The table summarizes the spectral positions and provides the biochemical attribution of each peak observed in the deconvoluted MR spectrum.

Nr.	ppm	Assignment
1	1.03	Lipids (alkyl chain CH_2_)
2	1.14	Lactate
3	1.87	*N*‐Acetylaspartate
4	2.3	Glutamate
5	3.21	Choline
6	3.40	Taurine
7	3.81	Creatine
8	4.72	H_2_O (water)
9	4.80	H_2_O (water)
10	5.35	Glucose

Different components of Cho and Cre were not separated, and the taurine peak overlaps with mI. The specific TEs employed in the MRS allowed us to choose taurine between the two; however, at this point, further investigations are necessary for better precision.

The good correspondence between the simulated and experimental spectra confirms the reliability of the AMARES fitting approach applied to retinal MR signals. All identified peaks were in agreement with expected chemical shift values reported in the literature for similar biological tissues. The signal‐to‐noise ratio (SNR) was sufficient to allow consistent fitting across samples, and no significant residuals were observed after subtraction of the fitted components. These results validate the use of jMRUI and the AMARES model as a robust methodology for metabolite quantification in retinal MR spectroscopy, enabling further analysis of metabolic alterations under different physiological or pathological conditions.

## 4 Discussion

### 4.1 Biological Interpretations

^1^H‐MRS enables the in vivo identification of alterations in retinal metabolites, demonstrating relatively low interindividual variability. The retinal environment seems stable, with only slight variations in lactate and, to a lesser extent, Glu metabolism. These variations could be explained by individual reactions to stress and variations in general metabolism (like heart rate and temperature). Acute metabolic stress (caused by handling, restraining, and response to general anesthesia) may produce decreased Glu or increased lactate levels [15].

Retinal ^1^H‐MRS investigation presents certain challenges, some of them related to the dimension of the tissue. Human and rat retinas, alike, have a thickness of only 200 μm, extended between *ora serrata* and the optic nerve, and applied onto choroidal tissue, from which is separated by the RPE. On the surface, the retina is coated with the vitreous body. In the rat eye, especially, the crystalline lens is large and is situated in the vicinity of the retina (Figure 1C). Unfortunately, it is not possible to scan the retinal tissue only. The voxel of interest will also contain choroid, vitreous body, and the periphery of the lens, the latter two structures with high‐water content (vitreous body up to 99%). However, we aimed to focus the detection onto the retina, at the posterior pole, in the central region, next to the optic nerve. Blue light exposure, on the other hand, is expected to produce stronger reactions in the most reactive tissue, which is the retina, while the surrounding tissues (lens, vitreous body, and choroid) remain mainly stable. Any changes in metabolites could be expected to arise from the retinal stimulation.

The resulting ^1^H‐MRS spectra acquired on rodents allowed for a precise deconvolution to identify key biomolecules and to compare these findings with the expected chemical shift values already described in the literature, regarding similar tissues (brain, in particular) [16]. The main chemical shifts were assigned for lipids, lactate, NAA, Glu, Cho, taurine, Cre, water, and glucose. Lipid identification in MRS refers to mobile intracellular triglycerides and cholesterols and has a strong peak at 1–1.3 ppm, corresponding to fat accumulations in brain and muscles. In ^1^H‐MRS of lipids, the main alkyl CH_2_ protons typically produce a large, characteristic signal (or set of overlapping signals) [17]. This is often the most intense lipid signal due to the large number of equivalent methylene groups in the long fatty acid chains. At the retinal level, the lipid peaks observed in this study at 1.03 mostly refer to alkyl chain CH_2_(*n*) of lipids. Short TE produces more prominent peaks and may partially overlap with lactate. Severe tissue damage with liberation of membrane lipids increases the lipid detection. Lactate [5] is usually seen as a peak at 1.33 pm, in the brain and especially in the cerebrospinal fluid. It is elevated in case of hypoxia or brain injury. NAA, a marker of neuronal integrity, is the largest peak, at around 2 ppm. It is synthesized in neurons and transported through axons, and it is decreased in brain tumors. An increase in NAA is seen in brain recovery. Glu–Gln complex (Glx) lies between 2.1 and 2.4 ppm and is changed in case of stroke and hypoxia. Glu is one of the primary excitatory neurotransmitters in the brain. Isolating Glu from Gln is difficult in MRS; therefore, most researchers report a Glx. Cho resonates at 3.2 ppm, it is an astrocytic membrane marker, and it is increased in brain tumors or multiple sclerosis. Cho is involved in pathways of phospholipid synthesis and degradation and reflects the integrity of membranes. Infarction or inflammation may lead to an increase in Cho levels [18]. Taurine resonates at 3.4 ppm and is usually elevated in medulloblastomas and may serve as an apoptotic marker in several cell types (fibroblasts, neurons, and T lymphocytes) or glioma tumors. The signal tends to overlap with Cho and mI, in low intensity ^1^H‐MRS. mI is a glial marker and is usually reported at 3.56 ppm. Residual water peaks at 4.7 ppm. Cre is usually seen at 3 ppm, with another peak at 3.9 ppm, and has a stable signal, serving as an internal reference point for MRS. Glucose has a peek at around 5.23 ppm, but glucose spectra are more broadly distributed in the band 3.1–5.3 ppm.

Regarding the visual system, the neurochemistry of the occipital lobe (V1 area, especially) is better researched. MRS [19, 20] of V1 in congenital blind populations found increased Cre, Cho, and mI, but variability in Glu change (Weaver saw no change, while Coullon saw an increase). An increase in mI may relate to an increase in glial cells. MRS investigations of the visual cortex in migraine observed variations in lactate (increased levels or no change), NAA (usually reduction or no change), and conflicting information regarding Glu/Glx levels [21].

MRS during visual stimulation revealed an increase in lactate and glucose. Long or short visual stimulation saw an increase in Glu of about 2%–4%, supporting a link between basic visual processing and Glu involved in neurotransmission and energy production [22]. GABA had no change.

Increasing the power of MRI leads to improved detection [23]. At 1.5 T, strong metabolic signals can be identified (NAA and Cho). At 7 T, the Glu complex is better detected. High‐energy magnetic fields allow the increase in SNR and the increase of the chemical shift. A long TR (in our case, TR = 1800 ms) minimizes signal attenuation. From 9.4 to 14.1 T, T1 relaxation time of metabolites in rat brain is around 1500 ms [24]. The PRESS sequence preserves all the magnetization available in the selected ROI. To suppress the undesired echoes, a long TE (TE = 16.5 ms) was used in our research.

Retinal metabolism is a particular one, involving one of the most active cells in the organism, the photoreceptors. The photoreceptor is an elongated polarized cell, the outer segment being highly enriched with phospholipid membranes and specialized to detect light. Most of the mitochondria are located centrally in the region called *ellipsoid* zone. High‐energy demand is linked to its incredible sensitivity to light (single photon being enough to stimulate the retina) and constant lipid production. Photoreceptors must react swiftly to major changes in light intensity (up to nine orders of magnitude). Glucose is delivered from the choroid and transported via the RPE (facilitated by glucose transporters) to the outer retina [25]. It is the primary fuel for photoreceptors, which then produce and export lactate to support the oxidative metabolism of neighboring cells (Müller glia and the RPE) via monocarboxylate transporter (MCT). Photoreceptors exhibit a high rate of aerobic glycolysis (similar to the Warburg effect in cancer cells), converting 80%–90% of the glucose they consume into Lactate, even in the presence of sufficient oxygen. This process is crucial for producing lipids and nucleic acids, necessary for the continuous renewal of the photoreceptor outer segments and for generating NADPH, which is essential for antioxidant defenses against light‐induced oxidative stress. It also generates ATP quickly, though less efficiently than aerobic glycolysis. Within the RPE and Müller cells, lactate is converted back to pyruvate and fully oxidized through the tricarboxylic acid (TCA) cycle and oxidative phosphorylation to produce a large amount of ATP to meet their own energy demands. The retina requires both glycolysis and oxidative phosphorylation to initiate vision [26].

Photoreceptors export lactate through MCT1 and MCT4, while RPE facilitates the bidirectional lactate exchange between photoreceptors and the choroidal circulation, thereby integrating outer retinal metabolism with systemic circulation through MCT3 [27]. This collaborative interplay between retinal cells (photoreceptors, ganglion cells, RPE, and Müller cells) constitutes the lactate shuttle, which represents a crucial metabolic pathway in retinal physiology, integrating energy transfer and metabolic signaling between cell types.

Lactate elevation is a biomarker of metabolic disfunction, but also a pathological mediator. It promotes angiogenesis by the activation of HIF‐1α, modulates immune response through protein lactylation, generates profibrotic signaling through transforming growth factor‐β. Excessive lactate shuttling restricts pyruvate entry into mitochondria, which reduces TCA cycle activity, aggravating mitochondrial stress [28].

NAA plays a role in neuronal health and function, acting as an acetyl‐group reservoir for lipid synthesis and an indirect regulator of mitochondrial energy production. NAA is synthesized in the neuronal mitochondria and functions as a critical reservoir of acetyl groups needed for the synthesis and maintenance of myelin lipids in glial cells. It is transported out of the neuron and into glial cells, where it is broken down by the enzyme aspartoacylase II (ASPA) into aspartate and acetate; the acetate is then used for lipid synthesis. Retinal NAA is produced through the mini‐Krebs cycle, an energy‐efficient metabolic pathway, primarily identified in retinal photoreceptor cells. This cycle enables cells to uncouple glycolysis from mitochondrial oxidative phosphorylation. It is fueled by anaplerotic substrates like Gln and branched‐chain amino acids, rather than glucose [29].

In intact biological tissues like the retina, molecular motions are anisotropic. This leads to line broadening in the NMR spectrum, resulting in a less resolved, broad envelope of peaks for the CH_2_ groups rather than a sharp singlet. The retina is one of the vertebrate tissues with the highest content in polyunsaturated fatty acids. Thus, the retina is highly enriched in docosahexaenoic acid (DHA; 22:6n‐3), which has six methylene‐interrupted double bonds [30]. This structure imposes specific conformational constraints and dynamics. The various CH_2_ groups in DHA are not all chemically equivalent, as their positions relative to the double bonds differ. This results in several overlapping CH_2_ signals across a range of chemical shifts, rather than a single, simple peak. The photoreactive product of visual pigment bleaching, all‐trans retinal (AtRAL), accumulates in photoreceptor membranes and interacts with the lipids. These interactions lead to the formation of bisretinoid condensation products (such as *N*‐retinylidene–*N*‐retinylethanolamine [A2E]), which can impact the structural properties and dynamics of the lipid alkyl chains and the overall membrane, contributing to retinal disorders [31]. Eye ^1^H‐MRS at 11.7 T allowed the detection of many metabolites which were previously described in brain MRS. In healthy adult rats, the major identifiable retinal metabolites were lactate, NAA, Cho, taurine, Cre, and Glu. After the initial measurements, the animals were exposed to high levels of blue light and rescanned for up to 48 h. Blue light, particularly 440 nm wavelength, can reach the retina, in contrast to UV light which is highly blocked by the cornea and crystalline lens. The changes in certain metabolites after blue light exposure could be considered as proof that the MRS signal originated from the retina. Eye ^1^H‐MRS at 11.7 T could not reliably identify a few other metabolites usually seen in the brain: mI or GABA, for example. This could be caused by a restriction in detection ability, a particularity of retinal tissue itself, or the noise introduced by surrounding tissues.

Blue light is an important mediator of light‐night cycles, and the retina contains sensitive photoreceptors (especially the blue cones and photosensitive retinal ganglion cells). High‐energy blue light penetrates the cornea and crystalline lens and reaches the retina, with potential photo trauma. In daily life, normal LED lighting and computers do not generate enough energy to affect the retina, even after long‐term exposures [32]. Blue light hazards occur when the eye is exposed to high intensity sources, like the sun, welder lamp, and operating microscope. Several mechanisms describe the changes occurring in the retina after blue light exposure. Photoreceptors and RPE cells loss could be explained through photomechanical damage (light at high intensity producing compressing forces and microbubble formation), photothermal damage (melanin in RPE and choroid absorbing the photons with the denaturation of molecules and formation of abnormal molecular linkage which lead to the loss of function of the cells), and photochemical damage (light absorption changes the electron state of certain molecules, which upon their consequent rearrangement eventually return to baseline, accompanied by the release of energy that allows the formation of reactive oxygen species [ROS]). Photoreceptor cell death could occur through light‐induced alterations of vitamin A (essential for photoreceptor function) and cell degeneration through lipid peroxidation, under increased ROS. Genetic and environmental factors contribute to cell death under phototoxicity, resulting mainly in apoptosis and possible necrosis [2]. There is a synergistic effect of blue light and A2E that exacerbates the photochemical damage causing the activation of inflammatory reactions (including the release of interleukin 1 [IL‐1], tumor necrosis factor‐α [TNF‐α], caspase‐1, and monocyte adhesion factor [MCP‐1]), DNA damage, inhibition of mitochondria with caspase protein activation leading to increased apoptosis, and inhibition of lysosome function. Additionally, vascular endothelial growth factor (VEGF) activation under blue light increases vascular permeability. Blue light produces retinal alterations like those seen in various ocular diseases: AMD, glaucoma, or diabetic retinopathy. Oxidative stress could appear even at low doses (1–20 J/cm^2^). Computer industry adapted to this evidence, providing better protection against blue light emitted from computer screens. Special types of glasses offer similar protection, while the intraocular lens implanted after cataract surgery may be yellow tinted for the same effect [33]. Blue light filter reduced apoptosis by 56%–89% and DNA damage by 57%–81% in LED‐exposed cells [12, 15, 34, 35].

Blue light exposure induced changes in the ^1^H‐MRS spectra. There is an increase in lipids, which may be consistent with lipofuscin changes proposed in retinal phototoxic injuries. Blue light may have an impact on lipofuscin accumulation and A2E‐mediated phototoxic effect. A2E is the key fluorophore excited by blue light, leading to ROS accumulation [36].

Apoptosis damage is linked to blue light and could be estimated by the observed reduction in NAA. There is a shift in the NAA peak, also, distributed towards lactate. There is an abrupt reduction in NAA 10 min after exposure, and a stabilization afterwards, with fluctuations. NAA remains reduced at 80 min, more stable for the next 24 h and presents a marked and continued diminution at 48 h.

Glu is decreased immediately after blue light exposure. From 20 min after exposure, there is a constant increase in Glu levels, up to 48 h.

Glucose has a reduction immediately post exposure and a stabilization afterwards. At 80 min, there is still a slight reduction, no change in the next 24 h, and a rehabilitation at 48 h.

Lactate peak is markedly increased post exposure, at 10 min, and continues to increase for the rest of the examinations. In the interval between 80‐min scan and 24‐h scan, there is a small reduction; then, lactate is increased again at 48 h.

These metabolic changes also prove that the retinas were successfully exposed to blue light. A high illuminance (6000 lx) was considered necessary to inflict maximum change, having no previous knowledge of the discrimination power for the MRS in the eye region. Rat eye has anatomic peculiarities. The amount of light that enters the rat eye is higher than in humans, giving the large acceptance angle (almost three times greater than that for the human eye), meaning that light can enter from a broader range of directions [37].

In the acute illumination, there is an increased damage in the RPE area and less damage in the cone area of the retina. RPE controls the transit of nutrients to photoreceptors and supports the visual cycle. RPE controls lipid photooxidation and oxidative stress in the retina, serving as a defensive system [35, 38]. Retinal photoreceptors are the biggest consumers of energy throughout the body, processing glucose, especially. Glucose levels are expected to fluctuate during metabolic crisis. Glucose from choroid passes through the RPE to retinal photoreceptor, where it is converted to lactate through aerobic glycolysis. Lactate is released into the interphotoreceptor matrix and at the level of RPE. Suppression of glucose consumption in the RPE (possible during blue light exposure) could result in an increased amount of glucose into the retina [39]. In Kanow study, the excess of lactate may be the trigger of increasing glucose availability at the retinal level.

### 4.2 Limitations

Our study was the first in vivo MRS investigation of the eye, focusing on the retina. The small ROI size (integrating a 200‐μm‐thick tissue in the center of the voxel) accounts for higher noise compared with that of brain imaging. Signal origin could not be limited to the retina only but also to the vitreous body, crystalline lens, or orbital tissue. There are quite a few challenges regarding the eye ^1^H‐MRS investigation: A small ROI means difficulties in alignment and low precision in repeated examinations and difficulties in identifying the source of the signal (retina or surrounding tissues). We tried to overcome the latter with blue light stimulation: The retina is the probable source of all metabolic changes after stimulation. Other difficulties rely on the shimming of volumes with many tissue interfaces, water suppression artifacts, and long measurement time.

On the other hand, there is no normative data so far to allow for the comparison of our data to other retinal studies in vivo, to our knowledge. MRS has historically been conducted only on individual eye tissues. A ^1^H‐MRS study [12] investigating retinal metabolites on 14.1 T analyzed retinal tissue harvested from human donors (postmortem) and identified 25 metabolites, including lactate, Glu, Cho, mI, taurine, and adenosine‐triphosphate (ATP). The analysis showed some biochemical differences between the vascular tissues (including retina) and the avascular tissues (cornea and lens), while the most differences were seen between the most distant structures, retina and cornea. Retina showed less ATP, but a higher content in lactate and taurine. Cho level was high in the retina, while it was not assigned in the lens.

Ex vivo exploration of the eyes revealed a similar spectrum to in vivo MRS, confirming the validity of the method. Avoiding the motion artifacts and background noise allowed a better resolution, but the results paralleled the in vivo study. Chemical shifts and peak shapes remained similar, with the main differences being quantitative rather than qualitative, as expected with the cease of active metabolism.

How does eye MRS compare with other methods? Raman spectroscopy was able to detect selective metabolites in the retina. Carotenoids were detected based on confocal resonance Raman microscopy, namely, zeaxanthin and lutein [40]. Surface‐enhanced Raman scattering with gold nanoprobes for dopamine triggers could allow dopamine detection with high precision [41]. Raman spectroscopy is mainly restricted to in vitro studies of retinas, with concerns regarding the safety of lasers, convenience of detection, and accuracy [42]. The data regarding characteristic neurometabolites in the retina are missing in Raman‐related studies, which, with few exceptions, focus on carotenoid detection (related to AMD mechanisms). In a study from 2021 [43], the authors built a prototype Raman analyzer by connecting a scanning laser ophthalmoscope to a spectrometer and were able to detect a decrease in NAA levels in patients with multiple sclerosis and a long‐term increase of Glu in the case of optic nerve inflammation [43]. In conclusion, retinal MRS has no comparative results of other noninvasive technologies investigating the retina.

Obviously, a much larger number of specimens must be investigated by eye MRS to create a normative database. Our study investigated only a small number of animals used for calibration and acquisitions. Identification of the metabolites was done by comparing data in literature with other tissues‐normative, mainly the brain (brain MRS being a well‐established procedure). There was no postmortem validation of the metabolites' change, keeping in mind that one of the reasons that ^1^H‐MRS might be valuable in retinal research is that it avoids the sacrifice of the animal.

### 4.3 Future Directions and Clinical Perspectives

MRI spectroscopy could become an early noninvasive marker of retinal damage. In this preliminary study, metabolic retinal changes were observed after acute exposure to blue light. We could safely investigate the animal retina for apoptosis (decrease in NAA) or increase in lactate and Glu. All animals survived the examinations, without complications due to MRI or general anesthesia.

It is prospected that MRS will serve as a valuable tool for the noninvasive in vivo monitoring of neuronal health, energy metabolism, and other cellular processes in the retina. Moreover, it could revolutionize ocular drug discovery by speeding up the development of pharmaceutical formulations intended for prevention or therapy, especially those which target the inner structures of the eye. The relative spectral changes of key biomarkers recorded on the posterior segments of the eye could be exploited for the in vivo monitoring of the therapeutic efficacy or the indirect pharmacokinetic profiling of the active pharmaceutical ingredients. The possibility of self‐referencing within the same living individual (one eye with, one without treatment) could significantly improve the sensitivity in detecting true drug effects, reducing the number of required animals aligned with the 3Rs principle (focusing on Replacement, Reduction, and Refinement) [44], and stronger within‐subject comparisons could improve statistical efficiency aiming to support claims of efficacy, safety, or exposure consistency. Nonetheless, high‐energy MRI is required for better discrimination of the metabolites. The present technology allows researchers to investigate long‐run retinal processes in animal models, without invasive procedures, with less ethical constraints by circumventing severe discomforts or harm and ultimately without the need of sacrificing the animal. Further research is needed to improve the accuracy of the detection or the location of certain metabolites. A better understanding of metabolic changes in the vitreous body and choroid is needed to allow precision of retinal detection and to avoid confounders.

Translation of the technology into humans will allow us to better understand mechanisms of the common eye diseases: AMD, glaucoma, and inflammation. Multimodal imaging could combine MRS to Raman, ocular coherence tomography, or fluorescein angiography to allow a complete understanding of the disease, from structural to vascular, and finally to biochemical levels.

## 5 Conclusions

Retina‐located ^1^H‐MRS at 11.7 T allows in vivo detection of metabolic changes induced by blue light exposure. The technique lends itself to the investigation of retinal neurodegeneration, oxidative stress, and light toxicity, paving the way for better functional and metabolic research in a spectrum of highly prevalent eye diseases, from AMD to diabetic retinopathy and glaucoma. It adds the advantage of being noninvasive, saving the lives of the animals involved in the research.

## Author Contributions

L.S., A.F., O.S., E.B., S.S., and S.C. conceived and designed the study. L.S. and A.F. are equal contributors to this work and designated as co‐first authors. A.F. performed the experiments and data acquisition. All authors equally contributed to data processing and analysis. All authors reviewed and approved the final version of the manuscript and agreed to be accountable for all aspects of the work.

## Funding

This study was (partially) funded by the Romanian Ministry of European Investment and Projects (MIPE) and by the Ministry of Research, Innovation and Digitalization (MCID), project codes SMIS 2014+ 127725, Contract No. 352/390028/23.09.2021, acronym project INSPIRE‐I and SMIS 2021+ 324771, Contract MIPE No. G‐2024‐71962/23.10.2024 and Contract MCID No. 390005/23.10.2024, project acronym INSPIRE‐II.

## Conflicts of Interest

The authors declare no conflicts of interest.

## Data Availability

The data that support the findings of this study are available from the corresponding author upon reasonable request.
